# Postmenopausal endometriosis presenting as a pelvic mass invading the colon

**DOI:** 10.1002/ccr3.3074

**Published:** 2020-09-26

**Authors:** Ariel Polonsky, Lance Bruck

**Affiliations:** ^1^ Department of Obstetrics and Gynecology Jersey City Medical Center Jersey City NJ USA

**Keywords:** endometriosis, malignancy, mass, postmenopausal

## Abstract

An invading mass in a postmenopausal female is usually suspicious of being cancerous. Even though, endometriosis should always be included in the differential since its surgical management involves significant morbidity and even mortality.

## Graphical abstract

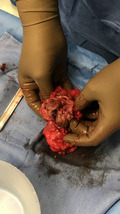

## 1 INTRODUCTION

Endometriosis is an estrogen‐dependent disease, which usually occurs in reproductive‐age females. The prevalence of endometriosis is around 6%‐10%.^1^ Even though endometriosis usually occurs in reproductive‐age females, it rarely occurs in postmenopausal women. This occurrence indicates the complexity of the disease since postmenopausal women generally do not produce estrogen and should not be widely affected.

## 2 CASE STUDY

**FIGURE 1:**
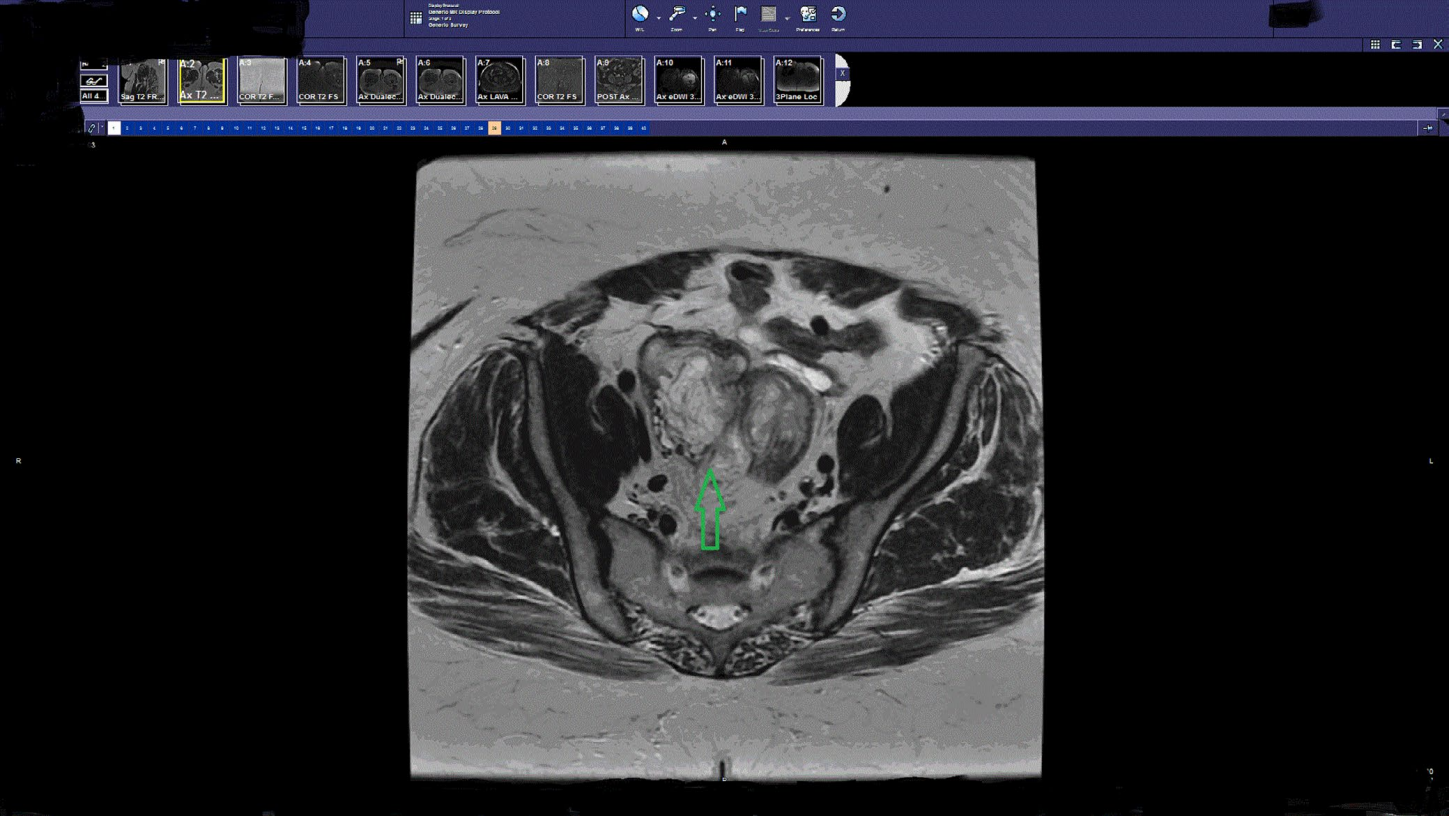
Pelvic MRI image demonstrating the pelvic mass invading into the sigmoid colon. The mass is marked with a green arrow

**FIGURE 2:**
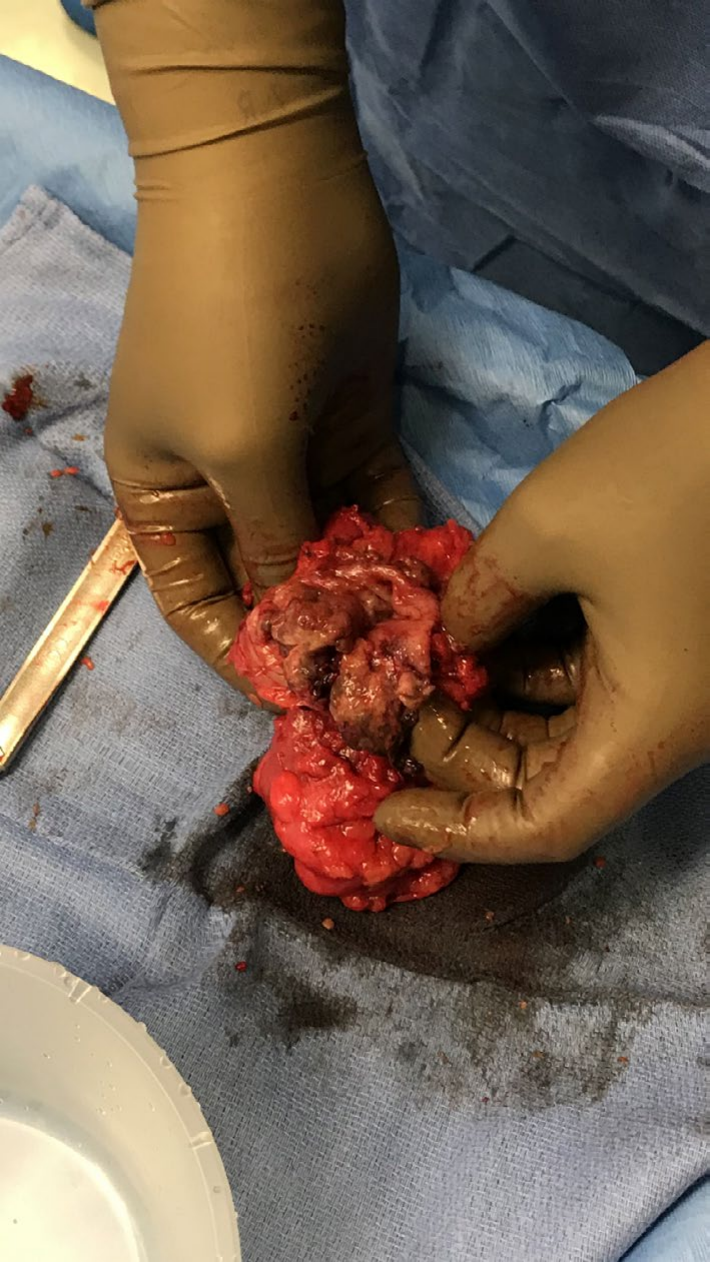
Intraoperative image of the sigmoid colon mucosa with the mass protruding through the mucosa

59‐year‐old woman presented to the emergency department complaining of acute rectal bleeding while denying previous occurrences. The only significant past medical history was essential hypertension. The patient's surgical history included the following: total abdominal hysterectomy 20 years prior due to a fibroid uterus and a left oophorectomy due to a large cyst. Physical examination was insignificant and stool occult blood test returned as positive. The patient had a CT of the abdomen and pelvic with contrast demonstrating a 7 × 6 × 5 cm pelvic mass surrounding and invading the sigmoid colon. MRI was additionally ordered confirming the CT findings (Figure 1). It could not be determined from the imaging modalities if the pelvic mass was invading the colon or arising from the colon. CA 19‐9 and CA‐125 were drawn and found to be abnormally elevated at 482 and 65, respectively. After considering all the findings, malignancy was suspected which included colon cancer or ovarian cancer. Colonoscopy was then performed obtaining multiple biopsies from the mass. The size of the mass was noted to be 4 × 4 cm with a heterogeneous abnormal contour. After an extensive pathological examination, the laboratory could not determine the origin of the tissue. The report described the biopsies as normal tissue with necrosis. As the patient was postmenopausal with an apparent invading pelvic mass, it was decided to proceed with a surgical excision. The surgery was complicated with extensive adhesions including the small bowel, large bowel, and the pelvic sidewall. After isolating the ureters bilaterally, the mass was reached deep in the pelvis and determined to be ovarian tissue on gross inspection. A biopsy was obtained and sent for intraoperative frozen section. The biopsy was reported as a benign lesion of undetermined significance. As the mass was invading into the colon, it was decided to perform an en bloc resection with a sigmoidectomy. The resection was performed and a colostomy raised as a result of severe inflammation and adhesions around the potential anastomosis site. (Figure 2). On postoperative day 2, the patient developed a severe ileus, which resolved with conservative management on postoperative day 7 with the assistance of nasogastric suction. Additionally, on postoperative day 7 the patient demonstrated signs of sepsis. On physical examination, the patient was diagnosed with a wound infection and a CT of the abdomen and pelvic ruled out an intraabdominal collection. The wound was opened, debrided, and cleaned with antibacterial packing. The patient was started on broad‐spectrum antibiotics, and a wound vac was applied to the open wound. On postoperative day 10, final pathology was reported as being invasive endometriosis with no malignant features. The patient was discharged on day 14 with a colostomy bag and a wound vac.

## 3 DISCUSSION

Deep endometriosis invading the bowel possesses a tremendous challenge and dilemma for the gynecologist. This is usually due to the high morbidity which involves surgical management. Intestinal involvement of deep invasive endometriosis is about 8%‐12% out of all endometriosis cases.^1^ The symptoms, which are usually expected with endometriosis such as dysmenorrhea, dyspareunia, chronic cyclic pelvic pain, and infertility, are not usually experienced in the postmenopausal state. These symptoms are usually expected to occur in a gonadal hormone‐rich environment. The ovary‐uterine hormonal cycle activates inflammatory pathways in endometriotic lesions in the pelvis resulting in all the symptoms mentioned above. During the postmenopausal state, the ovaries no longer produce estradiol; hence, the inflammatory pelvic environment evolves from other pathways, which are not well defined. In our case, the only presenting symptom was rectal bleeding. Even if endometriosis was known to exist in a postmenopausal patient, differentiating between a benign and a malignant lesion is a challenge. The pathological processes of both diseases can lead to distant metastasis and invasion of adjacent tissue. Imaging modalities may provide limited assistance secondary to similar appearance. Multiple studies were conducted on endometriosis with malignant transformation and determined that patients with endometriosis share a higher risk of overall cancer,^2^ specifically clear cell or endometrioid type. When an ovarian cyst is diagnosed in postmenopausal women, several criteria are used to try and predict its malignant potential. According to ACOG's practice bulletin number 174, cyst features which increase malignancy concerns include the following: size of 10 cm or more, papillary of solid components, irregularity, presence of ascites, and increased Doppler flow.^3^ Even though most of the features above were not present in our finding, what did increase our suspicions for a possible malignant lesion were the complex features of the cyst and colonic involvement. History of endometriosis was unknown in this patient, hence considering cyst features, patient age, colonic invasion, and tumor marker elevation, cancer probability was high and the surgical option seemed the most feasible one. In cases in which endometriosis has invaded the colon, three surgical options exist; the shaving method, the nodular resection method, and the segmental resection followed by end‐to‐end reanastomosis.^4^ The working diagnosis in our case was malignancy, and hence, the third option was used with a colostomy due to extensive local inflammation at the time of the procedure. When assessing the future of this patient, one should consider the possibility of recurrence. The incidence of recurrence in cases of colonic invasion in reproductive‐age women is about 16%.^5^ Medical treatment in the form of aromatase inhibitor or GnRh agonist might be attempted to prevent recurrence in this patient even though research data are lacking. Unfortunately, the benefit of these medications is unknown but the side effects are well known and should be considered before recommending any medical treatment.

## 4 CONCLUSION

Postmenopausal invasive endometriosis is a rare finding but should be considered in the differential of suspected pelvic malignancy in this group.

## CONFLICT OF INTEREST

None declared.

## AUTHOR CONTRIBUTIONS

Dr. Ariel Polonsky (corresponding author). Dr. Polonsky was responsible for managing the case and all its postoperative complications. He participated in literature review and initial manuscript writing. Dr. Lance Bruck (co‐author). Dr. Bruck was responsible for paper editing and review in addition to image selection.

## WRITTEN INFORMED CONSENT

Written informed consent was obtained from the patient for publication of this case report and accompanying images.
